# 4-Methyl-2,2-dioxo-1*H*-2λ^6^,1-benzothiazine-3-carboxylic Acid. Peculiarities of Preparation, Structure, and Biological Properties

**DOI:** 10.3390/scipharm86010009

**Published:** 2018-03-08

**Authors:** Igor V. Ukrainets, Ganna M. Hamza, Anna A. Burian, Svitlana V. Shishkina, Natali I. Voloshchuk, Oxana V. Malchenko

**Affiliations:** 1 Department of Pharmaceutical Chemistry, National University of Pharmacy, 53 Pushkinska st., 61002 Kharkiv, Ukraine; annahamza@ukr.net (G.M.H.); anna_chem@ukr.net (A.A.B.); 2 STC “Institute for Single Crystals”, National Academy of Sciences of Ukraine, 60 Nauki ave., 61001 Kharkiv, Ukraine; sveta@xray.isc.kharkov.com; 3 Department of Inorganic Chemistry, V. N. Karazin Kharkiv National University, 4 Svobody sq., 61077 Kharkiv, Ukraine; 4 Department of Pharmacology, N. I. Pirogov Vinnitsa National Medical University, 56 Pirogov st., 21018 Vinnitsa, Ukraine; voloshchuknatali@gmail.com (N.I.V.); malchenko2576@gmail.com (O.V.M.)

**Keywords:** esters, hydrolysis, 4-methyl-2,2-dioxo-1*H*-2λ^6^,1-benzothiazine-3-carboxylic acid, 2,1-benzothiazine, crystal structure, anti-inflammatory action, analgesic activity

## Abstract

In order to determine the regularities of the structure–analgesic activity relationship, the peculiarities of obtaining, the spatial structure, and biological properties of 4-methyl-2,2-dioxo-1*H*-2λ^6^,1-benzothiazine-3-carboxylic acid and some of its derivatives have been studied. Using nuclear magnetic resonance (NMR) spectroscopy and X-ray diffraction analysis, it has been proven that varying the reaction conditions using alkaline hydrolysis of methyl 4-methyl-2,2-dioxo-1*H*-2λ^6^,1-benzothiazine-3-carboxylate makes it possible to successfully synthesize a monohydrate of the target acid, its sodium salt, or 4-methyl-2,2-dioxo-1*H*-2λ^6^,1-benzothiazine. The derivatographic study of the thermal stability of 4-methyl-2,2-dioxo-1*H*-2λ^6^,1-benzothiazine-3-carboxylic acid monohydrate has been carried out; based on this study, the optimal conditions completely eliminating the possibility of unwanted decomposition have been proposed for obtaining its anhydrous form. It has been shown that 4-methyl-2,2-dioxo-1*H*-2λ^6^,1-benzothiazine is easily formed during the decarboxylation of not only 4-methyl-2,2-dioxo-1*H*-2λ^6^,1-benzothiazine-3-carboxylic acid, but also its sodium salt, which is capable of losing СО_2_ both in rather soft conditions of boiling in an aqueous solution, and in more rigid conditions of dry heating. The NMR spectra of the compounds synthesized are given; their spatial structure is discussed. To study the biological properties of 4-methyl-2,2-dioxo-1*H*-2λ^6^,1-benzothiazine-3-carboxylic acid and its sodium salt, the experimental model of inflammation caused by subplantar introduction of the carrageenan solution in one of the hind limbs of white rats was used. The anti-inflammatory activity and analgesic effect were assessed by the degree of edema reduction and the ability to affect the pain response compared to the animals of control groups. According to the results of the tests performed, it has been found that after intraperitoneal injection, the substances synthesized demonstrate a moderate anti-inflammatory action and simultaneously increase the pain threshold of the experimental animals very effectively, exceeding Lornoxicam and Diclofenac in a similar dose by their analgesic activity.

## 1. Introduction

**Figure 1:**
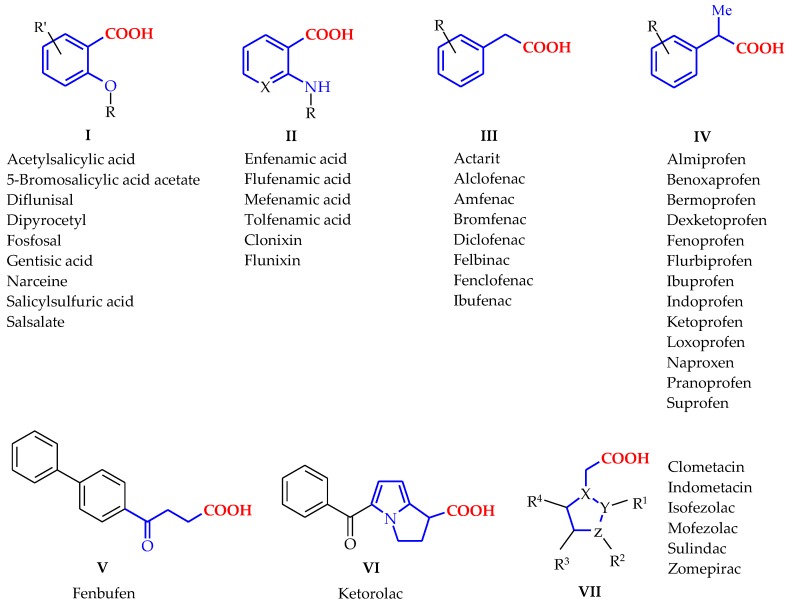
Analgesic-acids that are officially registered drugs [1,2].

Even a cursory review of reference works on drugs reveals the fact that in the arsenal of modern non-narcotic products against pain, the important role belongs to various carboxylic acids [1,2]. In accordance with the chemical structure, analgesic acids can be divided into several groups. First of all, it is, undoubtedly, derivatives of long-known salicylic acid **I** (Figure 1). A separate group is derivatives of anthranilic and 2-aminonicotinic acids **II** (X = C and N, respectively) that are structurally close to salicylic acid. Especially important and popular analgesics were created on the basis of phenylacetic acid **III**. Furthermore, the most representative group of analgesics officially permitted for use is comprised of 2-phenylpropionic acids **IV**. Derivatives of succinic acid (Fenbufen **V** and Suxibuzone), hetarylcarboxylic acid (Cinchophen, Etodolac and clinically important drug Ketorolac **VI**), and hetarylacetic acids **VII** (Indometacin, Sulindac, etc.) should also be mentioned.

These substances remain important despite the fact that the negative impact of substances with a free carboxyl group, first of all, on the gastrointestinal tract, is well known from medical practice. However, the presence of the acid fragment (or easily converted into it in vivo) is often reflected very positively on the biological properties exhibited by one or another molecule [3]. Therefore, carboxylic acids are always of particular interest for the search for new promising pain killers [4], and their possible side effects are eliminated by subsequent chemical transformation to labile non-acidic groups or by the special conditions of their administration [5].

**Figure 2:**
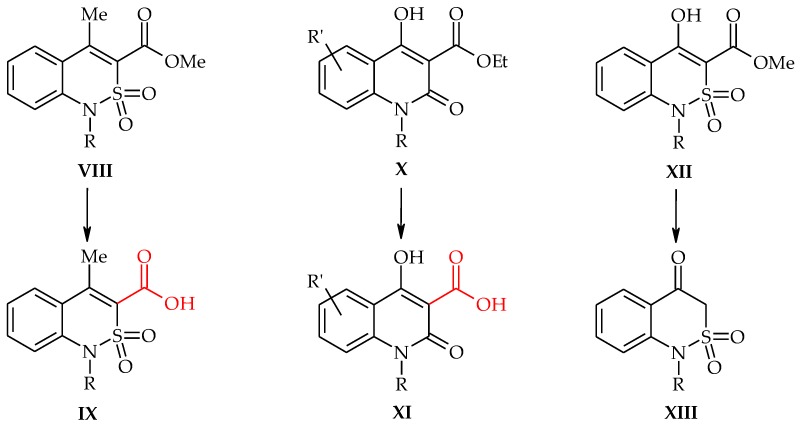
Probable (**IX**) and known (**XI** and **XIII**) hydrolysis products of esters of 4-hydroxy-2-oxoquinoline- [7] and 4-hydroxy-2,2-dioxo-2,1-benzothiazine- [8] 3-carboxylic acids.

Taking into account the above, it is of interest to study the structure and biological properties of 4-methyl-2,2-dioxo-1*H*-2λ^6^,1-benzothiazine-3-carboxylic acids **IX**. The main prerequisites for this research were the expressed analgesic activity demonstrated by their synthetic precursors — methyl-4-methyl-2,2-dioxo-1*H*-2λ^6^,1-benzothiazine-3-carboxylates **VIII** [6] as well as excellent results obtained during the transition from quinolone esters **X**, which are similar in terms of their structure, to acids **XI** [7] (Figure 2). Another incentive to study the behavior of 4-methyl-substituted benzothiazine esters **VIII** in alkaline hydrolysis conditions was a simple scientific curiosity: as is known [8], the corresponding acids cannot be obtained from their 4-hydroxy analogs **XII** since in the process of their formation, they are immediately decarboxylated to 4-oxo-3,4-dihydro-1*H*-2λ^6^,1-benzothiazin-2,2-diones **XIII**.

## 2. Materials and Methods

### 2.1. Chemistry

^1^Н- and ^13^С-NMR (proton and carbon nuclear magnetic resonance) spectra were acquired on a Varian Mercury-400 (Varian Inc., Palo Alto, CA, USA) instrument (400 and 100 MHz, respectively) in hexadeuterodimethyl sulfoxide (DMSO-*d*_6_) with tetramethylsilane as the internal standard. The chemical shift values were recorded on a *δ* scale and the coupling constants (*J*) in hertz. The following abbreviations were used in reporting spectra: s = singlet, d = doublet, t = triplet, m = multiplet. Melting points were determined in a capillary using a Electrothermal IA9100X1 (Bibby Scientific Limited, Stone, UK) digital melting point apparatus. The derivatographic study of 4-methyl-2,2-dioxo-1*H*-2λ^6^,1-benzothiazine-3-carboxylic acid monohydrate (**4**) was conducted using a Mettler TA 3000 (Mettler-Toledo GmbH, Bussigny, VD, Switzerland) thermoanalytical unit. The sample was heated in the inert gas atmosphere (argon, 20 mL/min), and the rate of heating was 5 °C/min. The synthesis of the starting methyl 4-methyl-2,2-dioxo-1*H*-2λ^6^,1-benzothiazine-3-carboxyate (**1**) was carried out by the method described in the study [6].

### 2.2. Sodium 4-Methyl-2,2-dioxo-1H-2λ^6^,1-benzothiazine-3-carboxylate Monohydrate (**3**)

Add sodium hydroxide (1.20 g, 0.03 mol) to the suspension of ester **1** (2.53 g, 0.01 mol) in 15 mL of H_2_O, then boil under reflux for 4 h. Cool, and acidify with HCl to рН 3.5. Allow to stand for 10 h at the temperature of 5–7 °C. Filter the crystalline sodium salt monohydrate **3** precipitated, and dry in air. Yield: 2.06 g (74%); after recrystallization from methanol, colorless crystals are obtained; melting point (mp) 190–192 °C (decomposition without melting); ^1^H-NMR (400 MHz, DMSO-*d_6_*): δ 11.45 (br. s, 1H, SO_2_NH), 7.50 (d, 1Н, *J* = 8.2 Hz, Н-5), 7.23 (t, 1Н, *J* = 7.6 Hz, Н-7), 6.96–6.89 (m, 2Н, Н-6,8), 2.33 (s, 3Н, 4-CH_3_). Analytically calculated (Anal. Calcd.) for C_10_H_8_NNaO_4_S • H_2_O: C, 43.01; H, 3.61; N, 5.02; S, 11.48. Found: C, 42.94; H, 3.53; N, 5.11; S, 11.40.

### 2.3. 4-Methyl-2,2-dioxo-1H-2λ^6^,1-benzothiazine-3-carboxylic Acid Monohydrate (**4**)

Obtained by a similar method—see the previous example. Dilute the reaction mixture to the volume of 10 mL, then acidify with HCl to рН 2.5–3.0. Yield: 2.26 g (88%); colorless crystals; mp 191–193 °C (decomposition, a double-end sealed capillary); ^1^H-NMR (400 MHz, DDMSO-*d_6_*): δ 11.82 (br. s, 2H, COOH + SO_2_NH), 7.76 (d, 1Н, *J* = 8.1 Hz, Н-5), 7.50 (t, 1Н, *J* = 7.6 Hz, Н-7), 7.23 (t, 1Н, *J* = 7.6 Hz, Н-6), 7.14 (d, 1Н, *J* = 7.9 Hz, Н-8), 2.47 (s, 3Н, 4-CH_3_, coincides with the signal of residual protons DMSO-*d_6_*). ^13^C-NMR (100 MHz, DMSO-*d_6_*): δ 162.9 (С=О), 145.0, 138.5, 132.7, 129.5, 128.4, 123.7, 121.6, 118.9, 17.8 (4-CH_3_). Anal. Calcd. for C_10_H_9_NO_4_S • H_2_O: C, 46.69; H, 4.31; N, 5.44; S 12.46%. Found: C, 46.75; H, 4.38; N, 5.39; S 12.54%.

### 2.4. 4-Methyl-2,2-dioxo-1H-2λ^6^,1-benzothiazine-3-carboxylic Acid (**5**)

Place 4-methyl-2,2-dioxo-1*H*-2λ^6^,1-benzothiazine-3-carboxylic acid monohydrate (**4**) (2.57 g, 0.01 mol) in a drying cabinet at the temperature of 100–110 °C for 10 h. Yield: 2.39 g (quantitative); colorless crystals; mp 210–212 °C (decomposition); Anal. Calcd. for C_10_H_9_NO_4_S: C, 50.20; H, 3.79; N, 5.85; S 13.40%. Found: C, 50.26; H, 3.83; N, 5.94; S 13.34%.

### 2.5. 4-Methyl-2,2-dioxo-1H-2λ^6^,1-benzothiazine (**7**)

- Add sodium hydroxide (1.20 g, 0.03 mol) to the suspension of ester **1** (2.53 g, 0.01 mol) in 15 mL of H_2_O, then boil under reflux for 20 h. Cool, and acidify the resulting aqueous solution of salt **6** with HCl to рН 3.5. Filter the precipitate of benzothiazine **7** obtained, wash with cold water, and dry in air. Yield: 1.66 g (85%); colorless crystals; mp 179–181 °C (ethanol); ^1^H-NMR (400 MHz, DMSO-*d_6_*): δ 11.31 (br. s, 1H, SO_2_NH), 7.22 (d, 1Н, *J* = 7.9 Hz, Н-5), 6.98 (td, 1Н, *J* = 7.6 and 1.4 Hz, Н-7), 6.61 (d, 1Н, *J* = 7.6 Hz, Н-8), 6.47 (t, 1Н, *J* = 7.4 Hz, Н-6), 6.07 (s, 1H, H-3), 2.12 (s, 3Н, 4-CH_3_). Anal. Calcd. for C_9_H_9_NO_2_S: C, 55.37; H, 4.65; N, 7.17; S 16.42%. Found: C, 55.46; H, 4.73; N, 7.10; S 16.36%.
- Keep 4-methyl-2,2-dioxo-1*H*-2λ^6^,1-benzothiazine-3-carboxylic acid (**5**) (2.39 g, 0.01 mol) at the temperature of 220-225 °C for 10 min. After СО_2_ evolution is stopped, cool the reaction mixture. Triturate the residue with 10 mL of cold ethanol. Filter the precipitate of benzothiazine **7**, and dry in air. Yield: 1.77 g (91%).
- Keep sodium 4-methyl-2,2-dioxo-1*H*-2λ^6^,1-benzothiazine-3-carboxylate monohydrate (**3**) (2.79 g, 0.01 mol) at the temperature of 200–210 °C for 15 min. The initially colorless salt **3** turns into a yellow powder without melting. Dissolve the resulting sodium salt **6** in 15 mL of H_2_O and acidify with HCl to рН 3.5. Filter the precipitate of **7**, wash with cold water, and dry in air. Yield: 1.81 g (93%).

The mixed samples of benzothiazine **7** obtained by different methods did not result in a depression of the melting point, and the ^1^H-NMR spectra of the compounds were identical.

### 2.6. X-ray Structural Analysis of Sodium 4-Methyl-2,2-dioxo-1H-2λ^6^,1-benzothiazine-3-carboxylate Monohydrate (**3**)

The crystals of sodium salt **3** (C_10_H_8_NNaO_4_S • H_2_O) were monoclinic, colorless. At 23 °C: *a* 5.241(2), *b* 30.040(8), *c* 7.119(1) Å; β 95.86(2)°; *V* 1114.8(5) Å^3^, *Z* 4, space group Р2_1_/c, *d*_calc_ 1.664 g/cm^3^, µ(MoK_α_) 0.341 mm^−1^, *F*(000) 576. The unit cell parameters and intensities of 7403 reflections (1962 independent reflections, *R*_int_ = 0.103) were measured on an Xcalibur-3 diffractometer (Oxford Diffraction Limited, Oxford, UK) using MoK_α_ radiation, a Charge Coupled Device (CCD) detector, a graphite monochromator, and ω-scanning to 2θ_max_ 50°. The structure was solved by the direct method using the SHELXTL program package (Institute of Inorganic Chemistry, Göttingen, Germany) [9]. The positions of the hydrogen atoms were found from the electron density difference map and refined using the “rider” model with *U*_iso_ = *nU*_eq_ for the non-hydrogen atom bonded to a given hydrogen atom (*n* = 1.5 for methyl, and *n* = 1.2 for the other hydrogen atoms). The structure was refined using *F*^2^ full-matrix least-squares analysis in the anisotropic approximation for non-hydrogen atoms to *wR*_2_ 0.204 for 1909 reflections (*R*_1_ 0.086 for 1280 reflections with *F* > 4σ (*F*), *S* 1.088). CCDC 1826303 contains the supplementary crystallographic data for this paper. These data can be obtained free of charge from the Cambridge Crystallographic Data Center [10].

### 2.7. X-ray Structural Analysis of 4-Methyl-2,2-dioxo-1H-2λ^6^,1-benzothiazine-3-carboxylic Acid Monohydrate (**4**)

The crystals of acid monohydrate **4** (C_10_H_9_NO_4_S • H_2_O) were monoclinic, colorless. At 23 °C: *a* 7.7755(7), *b* 10.1006(9), *c* 14.856(1) Å; β 100.533(3)°; *V* 1147.1(2) Å^3^, *Z* 4, space group Р2_1_/c, *d*_calc_ 1.391 g/cm^3^, µ(MoK_α_) 0.280 mm^−1^, *F*(000) 500. The unit cell parameters and intensities of 7106 reflections (1971 independent reflections, *R*_int_ = 0.036) were measured on a Bruker-Apex2 diffractometer (Bruker Corporation, Billerica, MA, USA) using MoK_α_ radiation, a CCD detector, a graphite monochromator, and ω-scanning to 2θ_max_ 50°. The structure was solved by the direct method using the SHELXTL program package (Institute of Inorganic Chemistry) [9]. The positions of the hydrogen atoms were found from the electron density difference map and refined in the isotropic approximation. The structure was refined using *F*^2^ full-matrix least-squares analysis in the anisotropic approximation for non-hydrogen atoms to *wR*_2_ 0.116 for 1913 reflections (*R*_1_ 0.045 for 1549 reflections with *F* > 4σ (*F*), *S* 1.127). CCDC 1826302 contains the supplementary crystallographic data for this paper. These data can be obtained free of charge from the Cambridge Crystallographic Data Center [11].

### 2.8. X-ray Structural Analysis of 4-Methyl-2,2-dioxo-1H-2λ^6^,1-benzothiazine (**7**)

The crystals of benzothiazine **7** (C_9_H_9_NO_2_S) were monoclinic, colorless. At 23 °C: *a* 12.793(2), *b* 4.9346(5), *c* 14.834(2) Å; β 105.06(1)°; *V* 904.3(2) Å^3^, *Z* 4, space group Р2_1_/c, *d*_calc_ 1.434 g/cm^3^, µ(MoK_α_) 0.321 mm^−1^, *F*(000) 408. The unit cell parameters and intensities of 8114 reflections (2624 independent reflections, *R*_int_ = 0.073) were measured on an Xcalibur-3 diffractometer (Oxford Diffraction Limited) using MoK_α_ radiation, a CCD detector, a graphite monochromator, and ω-scanning to 2θ_max_ 60°. The structure was solved by the direct method using the SHELXTL program package (Institute of Inorganic Chemistry) [9]. The positions of the hydrogen atoms were found from the electron density difference map and refined using the “rider” model with *U*_iso_ = *nU*_eq_ for the nonhydrogen atom bonded to a given hydrogen atom (*n* = 1.5 for methyl and hydroxyl groups, and *n* = 1.2 for the other hydrogen atoms). The structure was refined using *F*^2^ full-matrix least-squares analysis in the anisotropic approximation for non-hydrogen atoms to *wR*_2_ 0.129 for 2547 reflections (*R*_1_ 0.050 for 1482 reflections with *F* > 4σ (*F*), *S* 0.903). CCDC 1826304 contains the supplementary crystallographic data for this paper. These data can be obtained free of charge from the Cambridge Crystallographic Data Center [12].

### 2.9. Pharmacology

#### Anti-Inflammatory and Analgesic Test

All biological experiments were carried out in full accord with the European Convention on the Protection of Vertebrate Animals Used for Experimental and Other Scientific Purposes and the Ukrainian Law No. 3447-IV “On protection of animals from severe treatment” [13] (project ID 3410U14, approved 15 October 2015).

The biological studies were conducted on white Wistar male rats weighing 220–250 g. All experimental animals were under quarantine for 10 days. One day before the experiments, the animals were transferred to the research laboratory for adaptation. During the experiments, the animals were kept in standard conditions of the 12 h light-and-dark regimen with access to water and food ad libitum.

The anti-inflammatory activity of the compounds synthesized was studied on the model of the experimental inflammation process caused by subplantar introduction of 0.1 mL of 1% carrageenan solution [14,15] in one of the hind limbs of rats. The test compounds and the reference drugs were injected intraperitoneally into the animals of the experimental groups in 2 h after the introduction of carrageenan. In 3 h after the carrageenan injection (at the peak of inflammation), the volume of healthy and swollen limbs (mm^3^) was measured using a plethysmometer (Ugo Basile Biological Research Apparatus Company, Varese, Italy). The anti-inflammatory effect (in %) was assessed by the degree of edema reduction in the experimental animals compared to the animals of the control group. 

The analgesic activity was detected on the same model by determining the pain threshold—the smallest pressure force (g/mm^2^) on the rat’s foot, which caused a painful reaction of the animal expressed by localization of pain and/or paw withdrawal—the healthy and the injured limb. The measurements were performed using a baseline dolorimeter (Fabrication Enterprises, White Plains, NY, USA). The analgesic effect (in %) of the compounds studied was assessed by the ability to increase the pain threshold in the experimental groups compared to the animals of the control group.

All substances under research, Lornoxicam (Nycomed Austria GmbH, Linz, Austria) and Diclofenac (Hemofarm A.D., Vrsac, Serbia), were introduced intraperitoneally in the form of aqueous solutions or fine aqueous suspensions stabilized with Tween-80 at a screening dose of 20 mg/kg. The animals of the control group received an equivalent amount of water with Tween-80.

Seven experimental animals were involved to obtain statistically reliable results in testing each of the compounds **3** and **4**, the reference drugs, and the control. Processing of the results obtained was performed using STATISTICA 6.1 software package (StatSoft Inc., Tulsa, OK, USA). Descriptive statistics included calculations of the arithmetic means (M) and standard errors of the mean (±m). The significance of differences within one group was performed using Wilcoxon’s non-parametric test. The reliability of intergroup differences was determined using non-parametric Mann-Whitney *U*-criteria. Differences were considered to be statistically significant at *p* ≤ 0.05. 

## 3. Results and Discussion

### 3.1. Chemistry

**Scheme 1:**
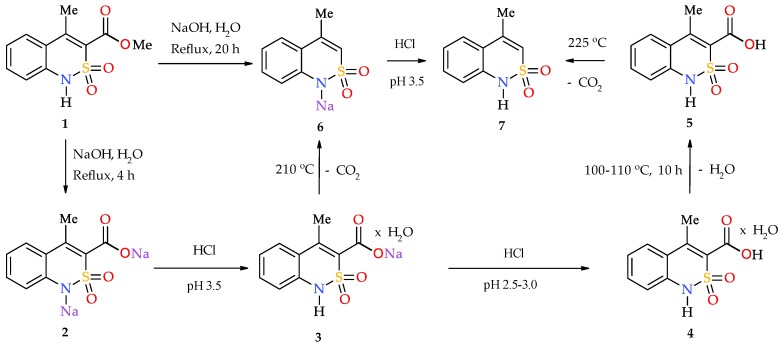
The peculiarities of 4-methyl-2,2-dioxo-1*H*-2λ^6^,1-benzothiazine-3-carboxylic acid (**5**) preparation.

A number of simple experiments conducted showed that methyl 4-methyl-2,2-dioxo-1*H*-2λ^6^,1-benzothiazine-3-carboxylate (**1**) in a boiling aqueous solution of sodium hydroxide was hydrolyzed quite easily and rapidly (Scheme 1). According to ^1^Н-NMR monitoring in 4 h after the beginning of hydrolysis, the initial ester in the reaction mixture was no longer detected. Unlike 4-hydroxy analogs **XII** [8], in this case, highly undesirable decarboxylation of hetaryl-3-carboxylic acid initially formed in the conditions of synthesis was not observed during this time. However, this does not mean that 4-methylsubstituted 2,2-dioxo-1*H*-2λ^6^,1-benzothiazine-3-carboxylic acids are not subjected to decarboxylation at all. As it turned out, this side process is typical for them too, and in relatively mild conditions. It is only necessary to increase the reaction time by several hours and decarboxylation becomes inevitable. For example, after the 20 h alkaline hydrolysis of ester **1,** exclusively 4-methyl-2,2-dioxo-1*H*-2λ^6^,1-benzothiazine (**7**) was isolated instead of the expected acid **5**.

**Figure 3:**
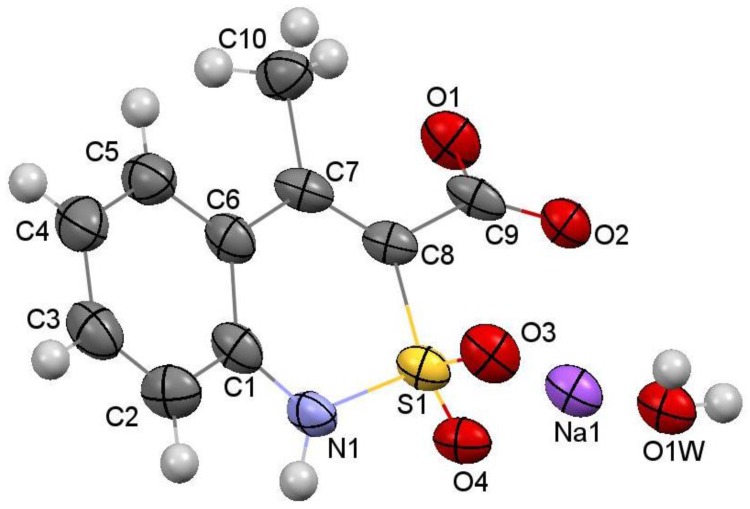
The standard representation of different atoms in different colors. The molecular structure of sodium salt monohydrate **3** with atoms represented by thermal vibration ellipsoids of 50% probability.

It is clear that the initial product of alkaline hydrolysis of ester **1**, regardless of the reaction duration, is always disodium salt **2**. But the subsequent attempt of the seemingly quite trivial transformation in acid revealed a number of interesting features. In particular, after acidifying the reaction mixture with hydrochloric acid to the usual pH for such cases (approximately 3.5), there was an abundant white precipitate of the presumable target acid **5**. As is known, the spatial crystal structure of derivatives of 2,1-benzothiazine-3-carboxylic acids largely determines their analgesic activity [16,17]. Hydrolysis of ester **1** into the corresponding acid from the very beginning was considered as a possible variant of strengthening analgesic properties. Therefore, it is quite natural that the resulting product was immediately subjected to X-ray structural analysis. In addition, it was found, quite unexpectedly, that this was not 4-methyl-2,2-dioxo-1*H*-2λ^6^,1-benzothiazine-3-carboxylic acid, but only its sodium salt, monohydrate **3** (Figure 3).

The anion negative charge of this salt is localized at the deprotonated carboxyl group. It is confirmed by the С_(9)_–О_(1)_ 1.261(7) Å and С_(9)_–О_(2)_ 1.248(7) Å bond lengths which are characteristic for the carboxylate anion (the mean value of such a bond length is [18] 1.255 Å).

The dihydrothiazine cycle of the studied anion adopts a conformation which is intermediate between twist-boat and sofa (the puckering parameters [19] are: S = 0.51, Θ = 44.8°, Ψ = 32.5°). The S_(1)_ and С_(8)_ atoms deviate from the mean plane of the remaining atoms of this cycle on 0.66 Å and 0.23 Å, respectively. The nitrogen atom has a planar configuration, and the sum of the bond angles centered at it is 360°. The carboxyl substituent is turned significantly relative to the С_(7)_–С_(8)_ endocyclic double bond (the С_(7)_–С_(8)_–С_(9)_–О_(1)_ torsion angle is −42.2(8)°), in spite of the formation of the С_(10)_–Н…О_(1)_ intramolecular hydrogen bond (Н…О 2.18 Å, О–Н…О 136°). The steric repulsion between the methyl group and atoms of the aromatic cycle is also obtained in the anion of the salt **3** (the shortened intramolecular contacts are Н_(5)_…С_(10)_ 2.53 Å and Н_(10а)_…С_(5)_ 2.71 Å compared to the van der Waals radii sum [20] 2.87 Å).

**Figure 4:**
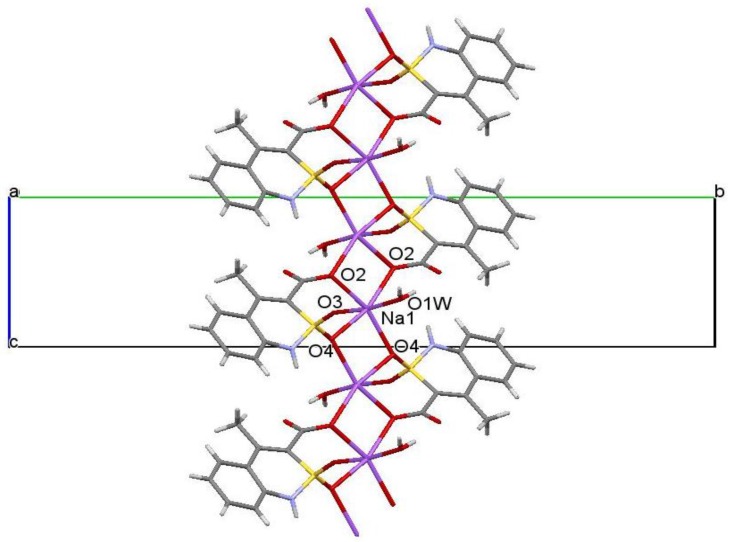
Packing of Sodium salt monohydrate **3** in the crystal phase. Projection along [1 0 0] crystallographic direction.

In the crystal phase, the anions of the salt **3** form columns along the [1 0 0] crystallographic direction. These columns are bound through sodium cations (Figure 4). The coordination number of the sodium cation is six. Each cation is bound with four anions, belongs to three different columns, and has additional coordination with a water molecule. The position of the water molecule is stabilized by the intermolecular hydrogen bonds (N_(1)_–H…O_(1w)’_ (−*x*, 1 − *y*, 2 − *z*) H…O 2.10 Å, N–H…O 132°; O_(1w)_–H_(1wa)_…O_(1)′_ (1 − *x*, 1 – *y*, 1 − *z*) H…O 1.83 Å O–H…O 173° and O_(1w)_–H_(1wb)_…O_(2)′_ (−*x*, 1 − *y*, 1 − *z*) H…O 2.00 Å, O–H…O 174°).

Sodium 4-methyl-2,2-dioxo-1*H*-2λ^6^,1-benzothiazine-3-carboxylate monohydrate (**3**) is readily soluble in water, and its unexpected easy release from the reaction mixture is most likely due to the salting out effect caused by the simultaneous presence of a rather large amount of sodium chloride and hydrochloric acid in addition to salt **3** in the solution. If necessary, this effect can be removed by simply adding a solvent, i.e., water. However, sodium salt monohydrate **3** is only dissolved. Additionally, for its transition into the acidic form, additional acidification of the reaction mixture to lower pH values of 2.5–3.0 is required (see Materials and Methods).

**Figure 5:**
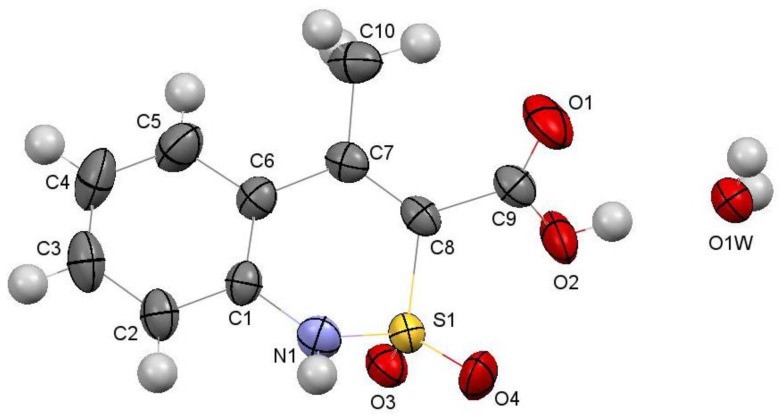
The molecular structure of acid monohydrate **4** with atoms represented by thermal vibration ellipsoids of 50% probability.

The X-ray diffraction study confirms the obtaining of the benzothiazine-3-carboxylic acid **4** and indicates that this acid exists as a monohydrate in the crystal phase (Figure 5). The molecular structure of the acid is very close to the structure of its anion in salt **3**.

The dihydrothiazine cycle of **4** adopts a conformation which is intermediate between twist-boat and sofa (the puckering parameters [19] are: S = 0.59, Θ = 55.9°, Ψ = 22.4°). The S_(1)_ and С_(8)_ atoms deviate from the mean plane of the remaining atoms of this cycle on 0.82 Å and 0.25 Å, respectively. The nitrogen atom has a planar configuration, and the sum of the bond angles centered at it is 360°. The carboxyl substituent is turned significantly relative to the С_(7)_–С_(8)_ endocyclic double bond (the С_(7)_–С_(8)_–С_(9)_–О_(1)_ torsion angle is 44.8(4)°), in spite of the formation of the С_(10)_–Н…О_(1)_ intramolecular hydrogen bond (Н…О 2.30 Å, О–Н…О 131°). The steric repulsion between methyl group and atoms of the aromatic cycle is also obtained in the acid **4** (the shortened intramolecular contacts are Н_(5)_…С_(10)_ 2.57 Å and Н_(10а)_…С_(5)_ 2.79 Å compared to the van der Waals radii sum [20] 2.87 Å).

**Figure 6:**
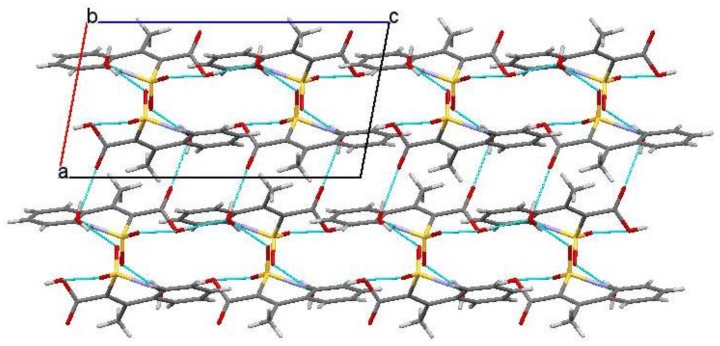
Packing of acid monohydrate **4** in the crystal phase. Projection along [0 1 0] crystallographic direction.

In the crystal phase, 4-methyl-2,2-dioxo-1*H*-2λ^6^,1-benzothiazine-3-carboxylic acid and water molecules are bound by the set of the intermolecular hydrogen bonds forming the three-dimensional net (Figure 6): N_(1)_–H…O_(1w)′_ (*x*, 0.5 − *y*, −0.5 + *z*) H…O 2.11 Å, N–H…O 175°; O_(2)_–H…O_(1w)_ H…O 1.82 Å, O–H…O 172°; O_(1w)_–H_(1wa)_…O_(1)′_ (−*x*, 1 − *y*, 1 − *z*) H…O 1.92 Å, O–H…O 168°; and O_(1w)_–H_(1wb)_…O_(3)′_ (1 − *x*, 1 − *y*, 1 − *z*) H…O 1.93 Å, O–H…O 167°.

**Figure 7:**
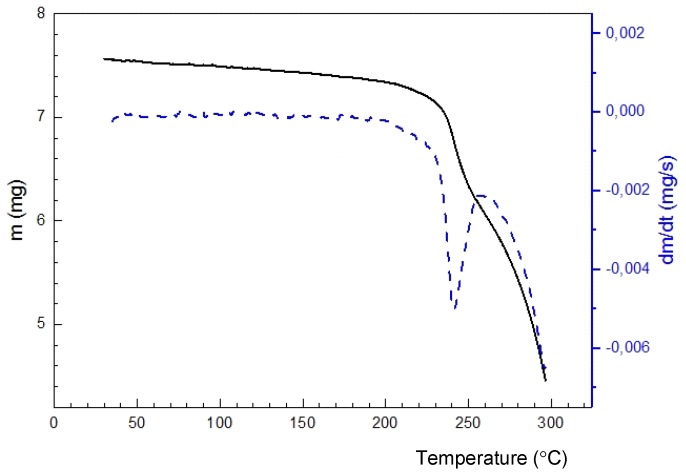
Derivatograms of acid monohydrate **4**: thermogravimetric curve (black); differential thermogravimetric curve (blue). Sample weight 7.57 mg.

The existence of 4-methyl-2,2-dioxo-1*H*-2λ^6^,1-benzothiazine-3-carboxylic acid in the form of monohydrate **4** does not matter fundamentally at this stage of our research. However, if this compound is used as a basis for further chemical transformations, the presence of crystallization water in its molecule can become extremely undesirable or even unacceptable. As a rule, the easiest and most available way to remove water from crystallohydrates is to dry them in different conditions, more often when heated. Therefore, in order to determine the thermal stability of acid monohydrate **4**, the derivatographic study was conducted under conditions of dry heating. The experimental data obtained (Figure 7) have convincingly shown that the smooth loss in mass begins immediately after the start of heating (see thermogravimetric curve); it obviously indicates the gradual removal of water. Otherwise, the test sample is sufficiently stable to a temperature of 200 °C; on the differential thermogravimetric curve at this temperature, there is a break that goes into the peak at 240 °C. The rapid loss in mass stops at 255 °C and then there is only uniform volatilization of the product. Within the temperature range from 200 to 255 °C, approximately 18% of the mass is lost, which uniquely corresponds to decarboxylation and transformation into 4-methyl-2,2-dioxo-1*H*-2λ^6^,1-benzothiazine (**7**).

Therefore, the derivatographic study conducted suggests that for the removal of crystallization water from acid **4** monohydrate and obtaining of anhydrous 4-methyl-2,2-dioxo-1*H*-2λ^6^,1-benzothiazine-3-carboxylic acid (**5**), a conventional drying oven at a temperature of 100–150 °C can be safely used.

Interestingly, 4-methyl-2,2-dioxo-1*H*-2λ^6^,1-benzothiazine (**7**) can be obtained not only by the long-term alkaline hydrolysis of ester **1** and thermal decarboxylation of acid **5** or its monohydrate **4**. Thus, initially colorless sodium 4-methyl-2,2-dioxo-1*H*-2λ^6^,1-benzothiazine-3-carboxylate monohydrate (**3**) when heated without melting rapidly turns into a powder of an intense yellow color, which appeared to be the sodium salt of 4-methyl-2,2-dioxo-1*H*-2λ^6^,1-benzothiazine (**6**), which, in turn, can be easily converted into the acidic form **7**.

**Figure 8:**
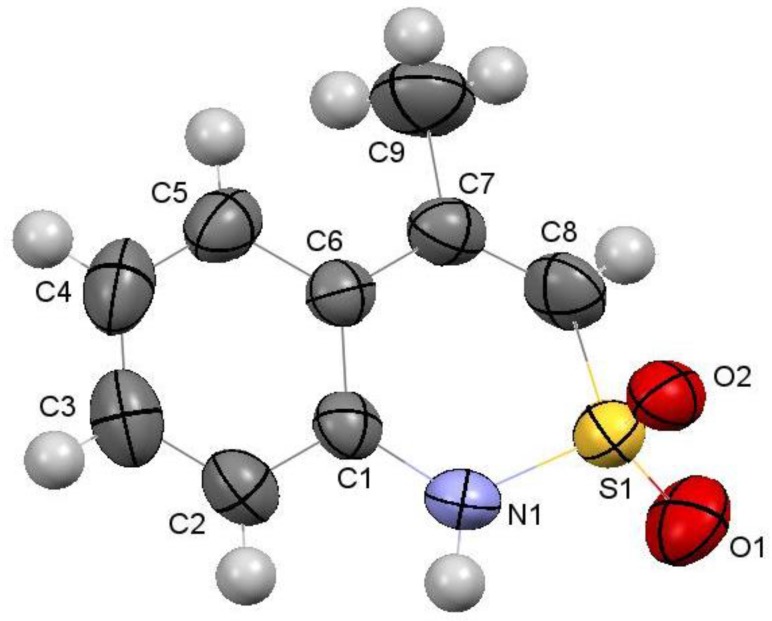
The molecular structure of benzothiazine **7** with atoms represented by thermal vibration ellipsoids of 50% probability.

Αccording to the X-ray diffraction data, the dihydrothiazine cycle of 4-methyl-2,2-dioxo-1*H*-2λ^6^,1-benzothiazine (**7**) (Figure 8) adopts a conformation which is intermediate between twist-boat and sofa similar to **3** and **4** (the puckering parameters [19] are: S = 0.50, Θ = 52.0°, Ψ = 17.7°). The S_(1)_ and С_(8)_ atoms deviate from the mean plane of the remaining atoms of this cycle on 0.67 Å and 0.17 Å, respectively. The nitrogen atom has a planar configuration, and the sum of the bond angles centered at it is 360°. The absence of the substituent at the С_(8)_ atom causes the significantly lower length of the С_(7)_–С_(8)_ bond (1.337(4) Å) compared to the substituted dihydrothiazines **3** and **4**. The steric repulsion between the methyl group and atoms of the aromatic cycle is obtained in **7** (the shortened intramolecular contacts are Н_(5)_…С_(9)_ 2.59 Å and Н_(9c)_…С_(5)_ 2.85 Å compared to the van der Waals radii sum [20] 2.87 Å).

**Figure 9:**
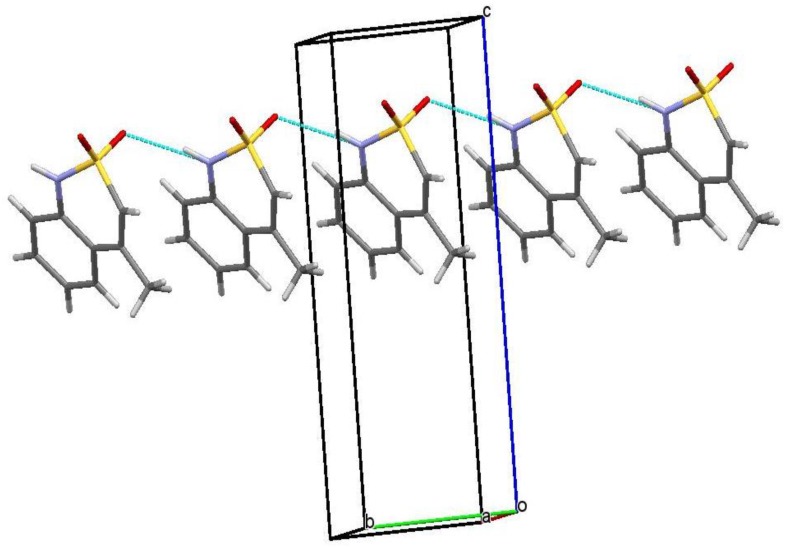
The chain of benzothiazine **7** molecules bound by hydrogen bonds.

In the crystal phase, the 4-methyl-2,2-dioxo-1*H*-2λ^6^,1-benzothiazine (**7**) molecules form chains along the [0 1 0] crystallographic direction (Figure 9) due to formation of the N_(1)_–H…O_(2)′_ (*x*, 1 + *y*, *z*) intermolecular hydrogen bond (H…O 2.09 Å, N–H…O 169°).

### 3.2. Evaluation of the Anti-Inflammatory and Analgesic Activity

The pharmacological tests conducted have shown that 4-methyl-2,2-dioxo-1*H*-2λ^6^,1-benzothiazine-3-carboxylic acid monohydrate (**4**) and its sodium salt **3** demonstrate moderate and statistically reliable anti-inflammatory properties on the carrageenan edema model in rats (Table 1).

Furthermore, the compounds synthesized deserve closer attention as potential analgesics and further study with the involvement of other experimental models and possible chemical optimization since they exceed Diclofenac and structurally similar Lornoxicam by the intensity of the analgesic effect (Table 2). The cause for the noticeable increase in its activity in sodium salt **3** compared to acid **4** can be its increased bioavailability. However, transition from derivatives of 2,2-dioxo-1*H*-2λ^6^,1-benzothiazine-3-carboxylic acids that are practically insoluble in water to their readily soluble forms due to salt formation is not always accompanied by an increase in analgesic properties [16].

## 4. Conclusions

The preparative method for obtaining 4-methyl-2,2-dioxo-1*H*-2λ^6^,1-benzothiazine-3-carboxylic acid has been proposed, which allows researchers to synthesize the target compound with a high yield and purity. It has been shown that as products of alkaline hydrolysis of methyl 4-methyl-2,2-dioxo-1*H*-2λ^6^,1-benzothiazine-3-carboxylate, both benzothiazine-3-carboxylic acid and its monohydrate, sodium salt, as well as 4-methyl-2,2-dioxo-1*H*-2λ^6^,1-benzothiazine, can be isolated. Using X-ray structural analysis, the peculiarities of the spatial structure of all compounds synthesized have been studied. The biological properties of 4-methyl-2,2-dioxo-1*H*-2λ^6^,1-benzothiazine-3-carboxylic acid and its sodium salt have been tested. In addition, a moderate anti-inflammatory activity of the substances studied has been determined and their high analgesic effect has been observed.

## Figures and Tables

**Table 1:**
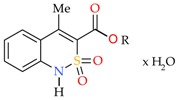
The Anti-Inflammatory Activity of Sodium Salt **3**, Acid **4**, and Reference Drugs.

Entry	Product	R	Volume of Damaged Extremity (mm^3^)	Volume of Non-Damaged Extremity (mm^3^)	ΔVolume (Volume Increase)	Anti- Inflammatory Activity, Compared to Control (%)
1	**3**	Na	516.3 ± 15.8	304.9 ± 14.0	211.4 ± 16.5 ^1,2,3^	48.9
2	**4**	H	568.6 ± 16.5	268.7 ± 7.59	299.9 ± 20.1 ^1,2,3^	27.5
3	Lornoxicam	–	360.5 ± 26.4	263.9 ± 19.8	96.58 ± 7.62 ^1^	76.7
4	Diclofenac	–	397.6 ± 11.9	306.6 ± 9.36	91.05 ± 5.52 ^1^	78.0
5	Control	–	768.7 ± 27.3	354.9 ± 11.6	413.7 ± 32.2	0

^1^ Differences statistically significant for *p* ≤ 0.05 vs. control. ^2^ Differences statistically significant for *p* ≤ 0.05 vs. Diclofenac. ^3^ Differences statistically significant for *p* ≤ 0.05 vs. Lornoxicam.

**Table 2:** The Analgesic Activity of Sodium Salt **3**, Acid **4**, and Reference Drugs.

Entry	Product	R	Pain Threshold on Damaged Extremity (g/mm^2^)	Pain Threshold on Non-Damaged Extremity (g/mm^2^)	ΔPain Threshold	Analgesic Activity, Compared to Control (%)
1	**3**	Na	350.0 ± 15.8	306.0 ± 19.6	44.0 ± 8.12 ^1,3^	86.2
2	**4**	H	294.0 ± 32.3	208.0 ± 17.7	86.0 ± 18.6 ^1^	73.0
3	Lornoxicam	–	441.0 ± 25.6	346.0 ± 23.4	95.0 ± 4.47 ^1,2^	70.1
4	Diclofenac	–	738.0 ± 18.3	679.0 ± 25.4	59.0 ± 9.27 ^1^	81.4
5	Control	–	593.0 ± 56.3	275.0 ± 32.1	318.0 ± 34.9	0

^1^ Differences statistically significant for *p* ≤ 0.05 vs. control. ^2^ Differences statistically significant for *p* ≤ 0.05 vs. Diclofenac. ^3^ Differences statistically significant for *p* ≤ 0.05 vs. Lornoxicam.
